# Links between Circadian Rhythms and Psychiatric Disease

**DOI:** 10.3389/fnbeh.2014.00162

**Published:** 2014-05-06

**Authors:** Ilia N. Karatsoreos

**Affiliations:** ^1^Department of Integrative Physiology and Neuroscience, Washington State University, Pullman, WA, USA

**Keywords:** biological rhythms, schizophrenia, depression, anxiety, bipolar disorder

## Abstract

Determining the cause of psychiatric disorders is a goal of modern neuroscience, and will hopefully lead to the discovery of treatments to either prevent or alleviate the suffering caused by these diseases. One roadblock to attaining this goal is the realization that neuropsychiatric diseases are rarely due to a single gene polymorphism, environmental exposure, or developmental insult. Rather, it is a complex interaction between these various influences that likely leads to the development of clinically relevant syndromes. Our lab is exploring the links between environmental exposures and neurobehavioral function by investigating how disruption of the circadian (daily) clock alters the structure and function of neural circuits, with the hypothesis that disrupting this crucial homeostatic system can directly contribute to altered vulnerability of the organism to other factors that interact to produce psychiatric illness. This review explores some historical and more recent findings that link disrupted circadian clocks to neuropsychiatric disorders, particularly depression, mania, and schizophrenia. We take a comparative approach by exploring the effects observed in human populations, as well as some experimental models used in the laboratory to unravel mechanistic and causal relationships between disruption of the circadian clock and behavioral abnormalities. This is a rich area of research that we predict will contribute greatly to our understanding of how genes, environment, and development interact to modulate an individual’s vulnerability to psychiatric disorders.

## Introduction

A significant problem that modern neuroscience aims to solve is the distress caused by neuropsychiatric disorders. The fundamental challenge is that these disorders are far from the unitary constructs we sometimes imagine, and almost certainly not caused by a single event, gene mutation, or neurotransmitter abnormality. Instead, these disorders are multifaceted neurobehavioral dysfunctions that in many cases also include symptoms outside the central nervous system. As such, neuroscience needs to address these challenges in an integrated fashion, leveraging the advances made using genetic, molecular, and physiological approaches. Several research groups are tackling the puzzle of neuropsychiatric disorders by exploring the hypothesis that homeostatic perturbations are at the root of such disease states. Understanding the mechanisms that maintain homeostasis and respond to environmental challenges that threaten homeostasis is of crucial importance. One such system is the circadian (daily) timing system, and studying how circadian rhythms are perturbed in psychiatric disorders may provide insight into their contribution to neurobehavioral changes in some mental disease.

This review will describe the function of the circadian timing system, discuss how various neuropsychiatric disorders such as depression, anxiety, and schizophrenia display disruptions in circadian timing, and present the hypothesis that in some cases these disorders may be triggered or exacerbated by a dysfunction in this crucial homeostatic system.

## Circadian Rhythms: A Brief Review

One of the most salient environmental signals available to organisms is the rotation of the Earth about its axis. The reliable and predicable circadian (daily) changes in light and temperature (to mention only a few variables) have provided organisms – from single-celled organisms to humans – a framework on which to temporally organize physiology. This framework allows organisms to accomplish two major tasks. The first task is predicting regularly repeating changes in the environment. Anticipating such changes in the environment can aid even the simplest single-celled photosynthetic organism in the prediction of daylight hours to optimize energy collection by allowing different biochemical pathways to become active at appropriate times. This then allows potentially incompatible biochemical processes to exist in their own temporal compartments, ensuring they do not interfere with each other. Equally as important is the adaptation to unanticipated or less periodic changes in the environment. The circadian system allows for stimuli in the environment to “phase shift” the endogenous clock, pushing it forward or backward, in order to adapt to changes in the outside world. Unfortunately, modern industrialized society can regularly produce light at the wrong times of day (e.g., light at night from electronics) that then can activate phase shifting processes inappropriately. This problem is exacerbated when individuals are chronically living “out of time” with their circadian clocks, such as shift workers, airline pilots, and medical workers to name a few. Growing evidence suggests that chronic circadian disruption can result in significant mental and physical health problems. However, the mechanisms by which disrupted circadian clocks lead to these health problems remain unknown. To determine potential pathways by which disrupted clocks can contribute to neuropsychiatric disease, we need to explore the processes that underlie circadian timing at the molecular and cellular levels.

Almost all biological processes in organisms with lifespans longer than 24 h display circadian rhythms. In more complex animals, the most obvious of these is the regulation of the rest–activity cycle. In mammals, the master circadian clock regulating nearly all circadian rhythms in the organism is located in the suprachiasmatic nucleus (SCN) of the hypothalamus. This neural structure contains a self-sustaining oscillator that synchronizes local clocks throughout the brain and body (Moore-Ede et al., 1984; Butler et al., 2009). These “peripheral” clocks are thought to set local time in many body tissues, and are hypothesized to allow optimal functioning by temporally organizing biochemical and cellular processes throughout the organism. Animal studies have shown that shifting the SCN clock by light causes an almost instant resetting of oscillators there, but oscillators in the rest of the body take numerous cycles to fully resynchronize to the SCN and the external environment (Yamazaki et al., 2000), the root cause of the general malaise associated with jet lag. The mechanisms by which this resynchronization occurs remain unclear, although numerous candidates have been suggested (Cheng et al., 2002, 2006; Buhr et al., 2010).

## How Does Circadian Disruption Affect Neurobehavioral Function?

Anecdotally, most of us are aware that disruptions in circadian timing through shift work, jet lag, or other processes can lead to neurobehavioral deficits. Such changes can manifest as alterations in mood, affect, or cognitive function. It should be noted that several of the most notorious industrial accidents in the past few decades, including the Bhopal disaster in India, the Chernobyl nuclear accident in Ukraine, and the Exxon Valdez oil spill in Alaska occurred during the night, with the individuals involved being shift workers of one sort or another. It is thought that several factors, including fatigue, interacted in each of these cases to cause or exacerbate the chain of events that lead to catastrophe (Colten and Altevogt, 2006). Thus, particularly in occupations with high cognitive loads, disrupted circadian clocks and sleep cycles could lead to significant degradation in cognitive function. An intriguing study in flight crews demonstrated that short recovery crews (those that are traveling mostly on transmeridian flights requiring repeated resynchronizations) showed decreased reaction times, increased error rates, and marked temporal lobe atrophy (Cho, 2001).

Animal models have also been applied to probe the connection between disrupted circadian clocks and neural and behavioral deficits. Gibson et al. (2010) used a repeated jet lag model in Syrian hamsters to explore the effects of chronic experimental “jet lag” on behavioral outcomes and neurogenesis in the hippocampus, since hippocampal neurogenesis is related to both cognitive and affective regulation, and may underlie depression (Samuels and Hen, 2011). They demonstrated that chronic jet lag by repeated phase shifting of the light–dark cycle results in learning and memory deficits accompanied by reductions in hippocampal neurogenesis. An important contribution of this study was the finding that deficits in hippocampal-dependent learning and memory persisted after cessation of the experimental jet lag (Gibson et al., 2010), suggesting that there may be long-lasting negative consequences of circadian disruption on brain function, even after the disrupting stimulus has been removed. In mice, Karatsoreos et al. (2011) demonstrated profound effects of circadian misalignment on the structure and function of prefrontal cortical neurons (Karatsoreos et al., 2011). Chronic (12 weeks) exposure to a shortened 20-h day (10 h light, 10 h dark) resulted in morphological changes in neurons in the medial prefrontal cortex (mPFC). Specifically, following circadian disruption neurons in layer II/III of the prelimbic mPFC had significant shrinkage of the apical dendrite, without observed changes in the basal dendrites. These gross changes were accompanied by simplification of the apical dendritic tree (Karatsoreos et al., 2011). Although the neural effects of the circadian disruption were stark, the behavioral effects were equally clear. Using a modified Morris Water Maze task that is sensitive to damage in the mPFC, circadian disrupted mice showed marked decreases in cognitive flexibility. In addition to the cognitive impairments, circadian disrupted mice demonstrated an “impulsive”-like phenotype, evidenced by entering a novel environment more quickly than controls. These findings were some of the first to experimentally link chronic circadian disruption to a reduction in the complexity of neurons that are important for attention, cognitive flexibility, and executive function. Although the mechanisms are still unknown, accumulating evidence supports a role for circadian disruption as a causative contributor to neurocognitive deficits.

## Links between Circadian Disruption and Psychiatric Disorders: Unfortunate Side Effect or Contributing Factor?

One of the most common, and highly disruptive co-morbid problems in many psychiatric conditions, including depression, obsessive–compulsive disorder, and schizophrenia, is disruption in the sleep–wake cycle. However, there is ample debate if these effects are merely *symptoms* of these disorders, or in fact, if they may be contributing causes.

Depressive disorders are characterized by multiple physiological and psychological symptoms, and present with circadian disruption in both behavior and in physiology. The disruption of the circadian clock can manifest as changes in sleep–wake cycles (Van Cauter and Turek, 1986; Turek, 2007), but growing evidence also shows circadian disruption at the level of the molecular circadian clock (Mendlewicz, 2009). Recent findings show that intensity of major depressive symptoms in humans is correlated with the misalignment of circadian rhythms (Emens et al., 2009), in that more severe depressive states are associated with the circadian pacemaker being more delayed relative to the timing of sleep onset. Whether this is a causal change is still unclear, but shift workers often suffer from mood disturbances and an increased risk for depression (Scott et al., 1997; Asaoka et al., 2013). It is important to consider that links between circadian function and depression might occur at many levels (Wirz-Justice, 2009). An interesting example of this multi-level interaction is evident in the development and use of agomelatine, a melatonin agonist that also has serotonergic activity. This drug is actively being used for its antidepressant actions, with significant results (de Bodinat et al., 2010). It is thought that agomelatine can also act as a circadian “resynchronizer” in models of depression (Morley-Fletcher et al., 2011; Koresh et al., 2012; Mairesse et al., 2013). In human studies, it has been demonstrated that agomelatine can increase the relative amplitude of circadian rhythms in the rest–activity cycle, including effects on sleep, which was accompanied by parallel improvement in depressive symptoms (Kasper et al., 2010). When taken as a whole, these findings suggest that circadian disruption may contribute to depression, though unraveling the etiology from symptomology can be difficult. Given that changes in hippocampal neurogenesis are observed following chronic circadian disruption, and that cell birth and proliferation in the hippocampus is related to mood and antidepressant efficacy (Gibson et al., 2010), it is evident that circadian disruption may contribute to the development or exacerbation of depressive disorders. As yet, how these various pathways interact and synergize is unknown, though changes in multiple interacting physiological systems induced by chronic circadian dysfunction are likely to be a precipitating factor. Although it is clear that there is a strong relationship between circadian disruption and depression, these effects are likely bidirectional.

In addition to cognitive deficits and depression, circadian rhythm abnormalities have also been explored in mania. It is well established that during manic episodes, sleep patterns are significantly altered (Wehr et al., 1983; Plante and Winkelman, 2008; Robillard et al., 2013), and circadian patterns of several physiological functions are attenuated (Goetze and Tolle, 1987; Souetre et al., 1988; McClung, 2007). To probe potential causative links between disrupted circadian clocks and mania, animal models must be leveraged. Several lines of evidence demonstrate that treating hamsters with lithium chloride (a potent pharmacological agent used to treat manic depressive disorders) significantly lengthens the period of their circadian clock (Terao, 1992; LeSauter and Silver, 1993; Klemfuss and Kripke, 1995; Iwahana et al., 2007). Detailed molecular work has shown that lithium treatment can alter several intracellular signaling cascades, including glycogen synthase kinase-3beta, a link to the circadian molecular clockworks (Iwahana et al., 2004; Padiath et al., 2004; Iitaka et al., 2005; Ko et al., 2010; Lamont et al., 2010; Osland et al., 2011). These studies suggest that this pharmacological treatment can reduce the symptoms of mania while also having direct effects on the circadian clock at both the cell/molecular level and the behavioral level. More recent work has begun to explore how defects in several key clock genes affect behaviors in mouse (McClung, 2011, 2013). Mutations in the core clock gene *Clock* can lead to mania-like behaviors (Roybal et al., 2007), and site-specific knockdown of *Clock* in the VTA can induce similar manic-like behaviors (Mukherjee et al., 2010). Together, the human and non-human animal models provide strong evidence that circadian dysfunction is not only a component of some forms of mania, but that altering the function of the molecular circadian clock can mimic many of these effects.

While pathways linking disrupted circadian clocks to cognitive function, depression, and perhaps even mania are being more clearly elucidated, links between circadian abnormalities and schizophrenia are less clear, both at the epidemiological and mechanistic levels. One reason for this lack of clarity is that the cause of schizophrenia remains elusive, and is likely a result of a combination of genetic and experiential factors. However, there are lines of evidence that point to strong links between disrupted circadian clocks and schizophrenia (reviewed in Jamadar et al., 2013; Monti et al., 2013). Epidemiological studies show that fragmented circadian rhythms, as measured by changes in rest–activity cycles or in sleep regulation, are observed in schizophrenic patients (Wirz-Justice et al., 1997, 2001; Wulff et al., 2006, 2012; Pritchett et al., 2012). This includes both sleep onset and sleep maintenance insomnia. Correlations have also been observed between the phasing of the melatonin rhythm and sleep in schizophrenia, and are commonly observed in many schizophrenic patients (Mills et al., 1977; Rao et al., 1994; Wirz-Justice et al., 1997). It is interesting to note that in most cases, the sleep/circadian effects observed in schizophrenia are *independent* of either the course of the disease or the pharmacological status of the patient (Monti et al., 2013). Several animal models are now being applied to attempt to gain a mechanistic handle on the interaction between circadian timing and schizophrenia. The “blind-drunk” (Bdr) mouse line, which presents schizophrenic-like symptoms (Jeans et al., 2007), has been shown to have phase-advanced (i.e., earlier starting) rest–activity cycles while also showing a fragmentation of their circadian cycles (Oliver et al., 2012). The Bdr mouse carries a mutation in the gene for synaptosomal-associated protein (Snap)-25 that leads to disruption of exocytosis. This points to an association between altered synaptic activity and neurobehavioral function observed in schizophrenia-like models and circadian rhythms. However, this work should be interpreted cautiously, as the effects of this mutation on circadian rhythms may have little to do with the effects of the mutation on schizophrenia-like behavior. It is more likely that rather than directly causing schizophrenia, disruption of the circadian clock may somehow alter susceptibility in individuals at risk of developing schizophrenia. Work by Vacic et al. (2011) shows that in humans, a copy number variant in the gene encoding for the receptor for vasoactive intestinal polypeptide that is found in the SCN (i.e., *Vipr2*) can result in an increase risk of developing schizophrenia (Vacic et al., 2011). As such, there is compelling and somewhat provocative evidence that disruption of the circadian clock may not only be a symptom of schizophrenia, but perhaps a contributing cause.

## Conclusion and Future Directions

The circadian timing system controls all physiological and behavioral rhythms, synchronizes them to the external environment, and ensures temporal isolation of incompatible physiological or behavioral processes (Kalsbeek et al., 2007; Karatsoreos and Silver, 2007; Butler et al., 2009). Thus, the circadian system sits at the center of a “web,” and can modulate the function of myriad physiological systems, both peripherally and centrally (Reppert and Weaver, 2002; Hastings et al., 2003). Since circadian rhythms are phylogenetically ancient, with many molecular components conserved between diverse species, from *Drosophila* to mouse to human (Bell-Pedersen et al., 2005), understanding how optimal functioning of this system contributes to fitness or vulnerability could have significant impact. That disrupted rhythms are observed in psychiatric conditions as diverse as depression, bipolar disorder, and schizophrenia (Mansour et al., 2005; Roybal et al., 2007; Mendlewicz, 2009; Cortesi et al., 2010; Sacco et al., 2010; Karatsoreos, 2012), makes it intriguing to hypothesize that they may play a role in their etiology. However, as this and many other reviews indicate, whether circadian disruption represents a symptom or an etiology is unclear, and the specific contributions of disrupted circadian rhythms to mental disease are poorly understood.

This review has presented several findings from both the human and non-human animal literature that support a role for disrupted circadian clocks in the etiology of mental disease. Since the causes of many of these neuropsychiatric disorders are multifaceted, it is unlikely that a single circadian mutation, or single instance of circadian disruption, would directly cause the development of a mental disorder. It is also important to note that while there is ample and growing evidence of a circadian contribution to many of the disorders discussed in this review, some of the evidence is indirect, and none of the evidence specifically obviates other causes for these neuropsychiatric diseases. It is our hope that this review provides an additional context to the already rich work on the genetic, developmental, and environmental etiologies of mental disorders. We hypothesize that disrupted circadian clocks may instead make individuals more *susceptible* to the development of neuropsychiatric disorders (Karatsoreos and McEwen, 2011, 2013). This effect may be in a manner similar to the stress-diathesis model, whereby environmental challenges have more severe outcomes due to underlying genetic or experiential differences (Morley, 1983). Thus, chronic circadian disruption through genetic abnormalities or environmental perturbation could make neural systems less able to cope with insults. This failure in resilience could lead to the onset of neuropsychiatric conditions in those individuals who are made more vulnerable because of other factors such as genetics, developmental experiences, or environmental exposures. While still conjecture, we feel that this is an exciting area for future research that will hopefully lead to great strides being made in understanding the complex causes of mental disorders.

## Conflict of Interest Statement

The author declares that the research was conducted in the absence of any commercial or financial relationships that could be construed as a potential conflict of interest.
